# Supplementary material for: Bayesian Weighted Sums: A Flexible Approach to Estimate Summed Mixture Effects
Source: Int J Environ Res Public Health. 2021 Feb 3;18(4):1373. doi: 10.3390/ijerph18041373 (PMC7913173; doi:10.3390/ijerph18041373)
Supplement: Supplementary file 1 [file ijerph-18-01373-s001.pdf]

International Journal of
Environmental Research and Public Health

MDPI

eSupplement 2: Traceplots for Table 3 model results.

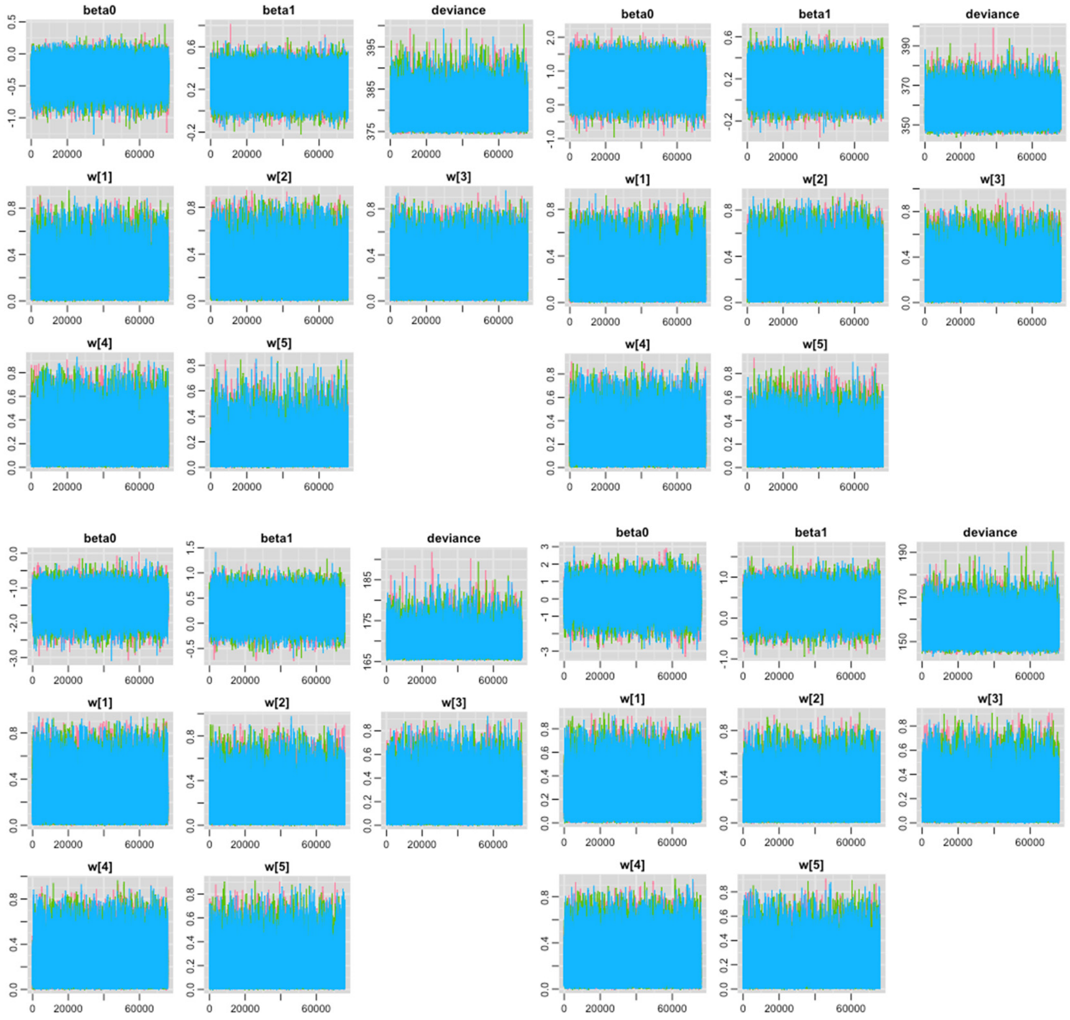

Figure S1. SRS crude model (**top left**), SRS fully adjusted model (**top right**), ASD crude model (**bottom left**), and ASD full model (**bottom right**). labels are described here: beta0 is the model intercept, beta1 is the summed exposure mixture estimate, and w[1] through w[5], in order, are weights for PBDEs 28, 47, 99, 100, and 153.

eAppendix: Description of likelihood function and approximate posterior distribution for a linear model, as used to estimate associations of exposure mixtures with SRS scores:

The likelihood for our model that uses a linear regression is:

$$L(\theta, w) = \prod_i N(y_i | \theta_0 + \theta_1(w_1 X_{1i} + w_2 X_{2i} + w_3 X_{3i} + w_4 X_{4i} + w_5 X_{5i}), \sigma^2)$$

The prior distribution on model parameters is:

$$\theta_0 \sim N(\theta_0 | 0{,}100)$$

$$\theta_1 \sim N(\theta_1 | 0{,}100)$$

$$(w_1, \dots, w_5) \sim Dirichlet(w_1, \dots, w_5 | \alpha_1, \dots, \alpha_5)$$

The posterior distribution is then written, up to a constant of proportionality, as:

$$f(\theta, w|y) \propto \left[\prod_i N(y_i | \theta_0 + \theta_1(w_1 X_{1i} + w_2 X_{2i} + w_3 X_{3i} + w_4 X_{4i} + w_5 X_{5i}), \sigma^2)\right] \times N(\theta_0 | 0{,}100) \times N(\theta_1 | 0{,}100) \times Dirichlet(w_1, \dots, w_5 | \alpha_1, \dots, \alpha_5)$$

Our results are derived from the posterior distribution which is unavailable in closed form and we use Markov chain Monte Carlo techniques to draw random samples from our posterior.
